# Identification of Lithology Based on Vibration Response of Drill Bit

**DOI:** 10.3390/s26196045

**Published:** 2026-09-24

**Authors:** Chong Wang, Qilong Xue, Zhengzhou Peng, Jin Wang, Weiguo Hai, Jun Qu

**Affiliations:** 1 Sinopec Huabei Oilfield Service Corporation, Zhengzhou 450006, China; wdlc282@163.com (C.W.);; 2 School of Engineering and Technology, China University of Geosciences, Beijing 100083, China

**Keywords:** laboratory experiments, vibrational differences, drilling parameter, lithology identification

## Abstract

The evaluation of rock properties is an indispensable part of the process of developing deep oil and gas resources. Traditional lithology identification methods have limitations such as high cost and time consumption. During the drilling operation, the interaction between the drill bit and the formation occurs, and the dynamic vibration response of the drill bit can represent the geological characteristics of the formation. In this paper, the differences in vibration response during the drilling process are explored by experimental methods, and the feasibility of using vibration signals to judge the change in rock stratum is investigated. A full-scale drill bit rock breaking test rig is constructed, and laboratory experiments on full-size Polycrystalline Diamond Compact (PDC) bit rock breaking are carried out, during which vibration response data under different drilling parameters are collected. The differences among experimental datasets are then analyzed and compared, and key data characteristics are extracted. Based on these, a new method for discriminating formation lithology is proposed. The results show that the amplitude of vibration response is closely related to drilling parameters and rock properties. The frequency composition of the vibration is only affected by the properties of the rock strata and is not affected by drilling parameters. Furthermore, the model established based on differences in vibration responses can effectively identify lithological changes, with an identification accuracy rate exceeding 95%. This study offers a theoretical basis for identifying lithologic changes in field production and serves as a valuable supplement to existing methods.

## 1. Introduction

When oil and gas resources are exploited, the recognition of the formation change law can provide an effective reference for drilling parameter optimization, hydraulic fracturing, drilling safety, etc. [1,2,3,4]. At present, the law of formation change is usually judged by the logging method and rock debris return, which have certain limitations [5,6,7]. In order to analyze BHA (bottom hole assembly) vibration during drilling, service companies have developed a near-bit vibration measurement tool. This tool provides a new way to obtain drilling bottom information [8,9]. During the drilling process, the drill bit is in direct contact with the rock layer. When the lithology being drilled changes, the interaction force between the bit and the formation also changes. At the same time, the vibration response of the bit changes accordingly. The use of vibration signals to identify the lithology can make up for the shortcomings of traditional logging methods, which are unable to measure the in situ stratigraphic stress.

At present, downhole vibration measurement devices have gradually matured. Kouzaiha et al. developed a downhole vibration measurement device mounted behind the drill bit, which enables high-frequency continuous measurement of parameters such as acceleration, weight on bit (WOB), rotational speed, and torque [10]. The vibration measurement device developed by Townsend et al. can be mounted on the side of the drill bit. It incorporates built-in instruments such as a triaxial accelerometer and a high-speed triaxial gyroscope. Its sampling rate can exceed 2 kHz, and its effective operating time is more than 200 h, allowing it to effectively measure downhole vibration data [11]. Huang et al. developed a probe-type intelligent bit parameter measurement device installed inside the drill bit, which is capable of measuring four parameters at the bit position: vibration, rotational speed, downhole temperature, and well inclination angle [12].

The development of downhole vibration measurement devices has provided favorable conditions for acquiring downhole vibration data. Accordingly, some researchers have conducted studies on how to utilize vibration signals to identify downhole conditions. Sitinjak believes that the vibration behavior of drilling tools is closely related to geological conditions. An analysis of near-bit vibration data from a well in Kuwait showed that when drilling through the shale formation to the carbonate formation, the carbonate formation generated stronger vibration than the shale section [13]. Flegner used a small drill bit to carry out the drilling test and used an acceleration sensor to collect the vibration acceleration of four rock types. Then the signal difference in different lithologies are analyzed [14]. Jones suggested a correlation between tangential acceleration and rock density and between tangential acceleration and gamma values. The higher the rock density, the higher the tangential acceleration; the lower the gamma, the higher the tangential acceleration [15,16]. Esmaeili suggested that drill string vibration is also influenced by formation characteristics such as uniaxial compressive strength. As the uniaxial compressive strength of the rock layer increases, the amplitude of vibration also increases, and vice versa [17]. Through an indoor test and theoretical simulation, Cai analyzed the changes in parameters such as torque and WOB of drilling tools under different conditions and believed that WOB, rotation speed and lithology are the main factors affecting amplitude fluctuation [18].

There is great complexity and uncertainty in drilling engineering [19]. It is a prerequisite for employing the vibration response to detect lithological variations that the effects of various factors on the vibration response are clearly established. Although it has been demonstrated by these studies that the vibration response changes correspondingly as drilling conditions evolve during bit penetration, the disparities between the impacts of lithological properties and those of other factors on drill tool vibration have yet to be clearly delineated. The rock breaking process is influenced by numerous factors. The resulting vibration response is difficult to capture accurately using mathematical and mechanical models. This remains a challenging endeavor [20,21].

In this paper, the disparities between the influences of lithological properties and those of other factors on drill tool vibration are examined, and a method of identifying rock layer types based on differences in vibration responses has been verified. The main research contents of this article are as follows: First, a full-scale test rig simulating the field drill string configuration was built for rock breaking. Secondly, multiple sets of rock breaking experiments with full-size Polycrystalline Diamond Compact (PDC) bit under different parameters are carried out. Subsequently, an analysis of near-bit vibration signals is performed, employing both time domain and frequency domain approaches. Finally, a formation lithology recognition model is developed based on vibration response discrepancies.

## 2. Experiments and Methodology

During the research process, the rock breaking test bench is built, and rock breaking experiments are carried out to collect data. A full-size PDC bit is used to drill into rock mass. The acceleration sensor is installed near the bit and collected vibration acceleration and rotational speed while drilling into three rock masses. These data are preprocessed. Then these data are analyzed in time domain and frequency domain to extract the vibration characteristics. At the same time, WOB and rotational speed are also considered. Then, BP neural network, random forest and GRNN are used to establish a rock discrimination model so as to recognize and analyze vibration signals. The research steps are shown in Figure 1 below.

### 2.1. Equipment Construction and Laboratory Experiments

To explore the response law of rock breaking vibration of the drill bit, it is very necessary to collect data near the drill bit. In field operations, a vibration measurement instrument can be installed at the back of the drill bit [22]. It collects vibration data during rotary drilling. In view of the difficulty of field test implementation and data richness, an experimental bench is built with reference to the actual drilling structure to obtain various measured data of the rock breaking process. The equipment can be used to collect vibration data, WOB, rotational speed and other parameters. Experimental equipment and rock samples are shown in Figure 2 below.

The rotational speed measuring device is used to measure the rotational speed of the power head applied to the drill pipe, and a flexible drill pipe is used to simulate the drill pipe in the field and transmit torque. The pressurized cylinders provide weight on bit. Two acceleration sensors are installed 0.5 m behind the drill bit. These acceleration sensors are used to measure the vibration acceleration and rotational speed during rock breaking and verify each other. WOB is measured by a pressure sensor mounted on the lower part of the rock sample.

To investigate the patterns of vibration response during rock breaking by the drill bit, the controlled variable method was adopted to adjust parameters such as formation properties, drilling pressure (WOB), and rotational speed, and multiple sets of experiments were conducted. Three kinds of rocks with different strengths are selected as experimental samples in the laboratory experiments. Three rock samples of granite, sandstone and limestone are drilled during the test. The rock sample size is 300 mm × 300 mm × 300 mm. A PDC bit with a diameter of 154 mm is selected. During the experiment, the sampling frequency of the acceleration sensor is set to 2 kHz. The sampling frequency of the rotational speed measuring device and the pressure sensor is set to 10 Hz. When the rotational speed is 22 r/min and the drilling pressure is 0–25 kN, the data collected during the experiment are shown in Figure 3 below.

### 2.2. Data Preprocessing and Analysis

Through drilling experiments, the vibration response data of the drill bit are collected. In addition, the rock type, WOB, and rotational speed were varied to investigate their effects on the vibration response. The experimental parameters are summarized in Table 1. In this section, time domain and frequency domain analytical approaches are used to explore differences in drill bit vibrations.

To analyze the relationship between formation type and vibration, the WOB and rotational speed are kept relatively stable during the experiment. In the experiments, rotational speed is maintained at 40 r/min and weight on bit at 15 kN. The rock samples are sandstone, limestone, and granite respectively. Tangential acceleration signals are shown in Figure 4 below. Here, the tangential acceleration is analyzed as an example. The subsequent analysis follows this rule. When drilling into granite, the vibration acceleration at the drill bit is approximately −6.03~5.91 g. When drilling into limestone, the vibration acceleration at the drill bit is approximately −6.69~4.63 g. The vibration acceleration is the smallest when drilling into sandstone, and the amplitude is about −4.06~3.88 g.

To further study the differences and characteristics of the vibration signals, the acceleration data are transformed into the frequency domain by means of the Fast Fourier Transform. The spectrum diagrams of the three vibration signals are shown in Figure 5. There are obvious differences in the frequency composition of the three groups of vibration signals.

With a constant WOB of 20 kN and rotational speeds of 20, 40, and 60 r/min, the tangential acceleration signal generated when drilling granite is shown in Figure 6 below. At a rotational speed of 20 r/min, the vibration acceleration was approximately ±6.1 g, and it reached around ±6.9 g when the speed increased to 40 r/min. When the speed is 60 r/min, the vibration acceleration is about ±10.1 g. The Fourier transform is employed to explore the frequency characteristics of the vibration signal. The spectrum diagrams of the three vibration signals are presented in Figure 6d–f. It is observed that raising the rotational speed scarcely changes the spectral characteristics of the acceleration signal, while the amplitude at the dominant frequency is increased.

To analyze the effect of WOB on the drill bit vibration response, triaxial acceleration data are collected during granite drilling. Rotational speed is held constant at 40 r/min, while the WOB is 10, 14, and 18 kN. The corresponding tangential acceleration signals are shown in Figure 7 below. When the weight on bit is 10 kN, the tangential acceleration is −3.24~3.45 g. When the weight on bit is 14 kN, the tangential acceleration is −4.69~5.26 g. When the weight on bit is 18 kN, the tangential acceleration is −6.41~7.49 g. As shown in the figure, tangential acceleration increases with increasing WOB. The spectrum diagrams of the three vibration signals are shown in Figure 7d–f. This result shows that the increase in WOB hardly changes the spectral characteristics of the acceleration signal. It leads to an increase in the amplitude at the main frequency.

### 2.3. Characteristics of Vibration Response

To quantify the differences between vibration signals, the characteristic values shown in Table 2 are extracted [23,24]. Experimental data from drilling in different rock samples are preprocessed. Characteristic values of triaxial acceleration signals and rotational speed are then extracted from the processed data. These features serve as the data foundation for constructing a rock-type classification model.

The value ranges of the feature values differ significantly. To facilitate comparative analysis, the feature values are normalized. In addition, box plots are used for statistical analysis to clearly illustrate differences in vibration data characteristics. The box plots of the normalized features for the three rock samples are shown in Figure 8.

Box plot comparison shows significant differences in vibration signal characteristics among rock types with different lithologies. The time domain characteristics of the three rock types are clearly distinct. Granite exhibits higher peak-to-peak value, root mean square value, and clearance factor than the other two rock types, while limestone and sandstone show smaller differences in these three features. The kurtosis index effectively distinguishes limestone from sandstone, as well as granite. Frequency domain features also exhibit significant differences. For example, when drilling granite, the low percentage of power in the frequency band of the vibration signal is higher than that observed in sandstone. Analysis of the characteristic values shows that a single value cannot fully capture the differences among rock samples. Therefore, multi-dimensional characteristic parameters are selected and combined with a neural network model to establish a method for classifying rock types.

The data characteristics of axial and tangential accelerations generated by drilling into three rock types are used to analyze the pattern of lithological changes. WOB and rotational speed have a significant influence on vibration signals, so they are also selected as input parameters. The t-SNE algorithm is used to analyze the data characteristics. t-SNE is a nonlinear technique for dimensionality reduction and data visualization. It maps data points from a high-dimensional space to a two- or three-dimensional embedding. This enables direct visual inspection of the structure of high-dimensional data. t-SNE is widely used to visualize clustering results [25].

The results are shown in Figure 9. Using the extracted data features, the three rock samples can be clearly distinguished.

### 2.4. Establishment of Rock Mass Discriminant Model

Artificial intelligence is widely used in oil drilling and mining resource extraction because of its robustness and accuracy [26,27,28]. Due to the complexity of drilling conditions, the input data features and rock types exhibit mostly nonlinear relationships, and artificial intelligence algorithms are thus employed to establish the lithology recognition model.

(1) Back propagation (BP) neural network

As one of the most widely adopted neural network architectures, the back propagation (BP) neural network has been extensively applied in various fields [29]. The process of neural network training includes: (1) Initialize the network parameters, including connection weights and node thresholds. (2) Compute the outputs of the hidden layers by forward propagation of the input signals. (3) Calculate the final output of the BP neural network. (4) Calculate the prediction error by comparing the network output with the expected target. (5) Update the connection weights based on the error gradient. (6) Adjust the node thresholds according to the prediction error; (7) Determine whether the iteration termination condition is satisfied; if not, return to step (2). The algorithm structure is shown in Figure 10.

(2) Random forest algorithm

Random forest is widely used in classification recognition because of its high accuracy, resistance to overfitting, and ability to handle large numbers of features and data [30,31]. As an ensemble learning method, the random forest algorithm generates a classifier that aggregates the outputs of numerous decision trees. When these input data are fed into the model, each decision tree independently carries out classification. The model’s final outputs are decided by majority voting across the ensemble. The diagram of the random forest algorithm is illustrated in Figure 11 below.

The process of the random forest algorithm includes the following steps: (1) the Bootstrap method is applied to resample the original data, randomly generating T training sets; (2) For each sub-data set, a decision tree is constructed; (3) Each decision tree is allowed to grow completely without pruning; (4) For each test sample, all decision trees perform classification, and their respective outputs are obtained; (5) The outputs of all decision trees are integrated by majority voting to determine the final classification result.

(3) Generalized Regression Neural Network (GRNN)

The Generalized Regression Neural Network (GRNN) offers notable advantages, such as strong nonlinear mapping capability, high fault tolerance, and robust performance in the presence of noisy or limited data [32]. The algorithm is suitable for solving nonlinear problems and delivers better predictions when the sample data are small [33]. The structure of the algorithm is depicted in Figure 12.

The steps to build the GRNN algorithm are as follows: (1) Determine the centers of the radial basis functions. (2) Set the smoothing parameter. (3) Determine the connection weights that link the pattern layer to the summation layer. (4) Perform neuron computation in the output layer.

## 3. Results and Discussion

In order to verify the feasibility and universality of the method to discriminate the lithology classes by the vibration signals, three machine learning methods are employed to build the lithology identification models, and their identification accuracies are compared. The vibration data of drilling sandstone, limestone and granite under various conditions are selected. The total sample size is 1403. Among all samples, 70% are used for model training and 30% for model validation. The corresponding lithologies are encoded by the one-hot method to distinguish different lithology classes.

In this section, BP neural network, random forest and GRNN are used to analyze the data characteristics and rock strata data respectively, and three different identification models are established. Table 3 lists the parameter settings for the three algorithms used during model establishment.

The confusion matrix is used as a measure of the accuracy of the model. Diagonal cells correspond to correctly categorized observations. Each cell usually shows two values: the absolute sample count and its percentage of the total validation dataset. Overall accuracy is the most common classification metric. It measures the proportion of correctly predicted samples in the test set. A higher accuracy generally means better model performance.

The BP neural network is employed to build a reliable prediction model. A total of 983 labeled data samples are selected as the training dataset, which is used to iteratively optimize the network parameters. Once the model training phase is finished, 420 independent data samples are selected as the test set. As shown in the confusion matrix of the test set in Figure 13a, the x-axis and y-axis represent the true labels and the predicted labels, respectively. The model achieves a recognition accuracy of 97.4%. There are 71 samples of sandstone with 95.8% recognition accuracy, 236 samples of limestone with 99.6% recognition accuracy, and 113 samples of granite with 93.8% recognition accuracy.

The random forest algorithm is employed to perform data analysis on the same sample set. The test dataset contains 420 samples from three rock types. The corresponding recognition outcomes are illustrated in Figure 14 below. In this confusion matrix, each cell contains two values: the top number represents the absolute number of samples, and the bottom percentage indicates its proportion of the total test samples. Taking the diagonal entry of granite as an example, the numbers ‘101/24.0%’ signify that 101 granite samples are correctly identified, accounting for 24.0% of the entire dataset. The model achieves a recognition accuracy of 96.4%. There are 71 samples of sandstone with 97.2% recognition accuracy and 236 samples of limestone with 99.6% recognition accuracy. For granite, 113 samples are evaluated, yielding a recognition accuracy of 89.4%. The detailed category-level recognition results are displayed in Figure 14b.

The GRNN is applied to perform data analysis on the same dataset. The corresponding recognition outcomes are illustrated in Figure 15 below; the model achieves a recognition accuracy of 95.2%. From the confusion matrix presented in Figure 15a, the recognition accuracies are 87.3% for 71 sandstone samples, 97.5% for 236 limestone samples, and 95.6% for 113 granite samples. It can be observed that sandstone suffers relatively more misclassification errors compared with limestone and granite, which may be attributed to overlapping feature characteristics between sandstone and the other two lithologies. The detailed category-level recognition results are displayed in Figure 15b. Most samples can be correctly assigned to their respective rock categories, and sporadic vertical deviations correspond to the misclassified cases reflected in the confusion matrix.

Due to its direct contact with the rock formation, the drill bit can promptly detect changes in lithology. As downhole vibration measurement devices continue to mature, using vibration signals to evaluate lithological properties enables real-time identification of the lithology being drilled. This approach can compensate for the limited real-time capability of existing logging-while-drilling (LWD)-based lithology evaluation. In addition, near-bit vibration signals can be acquired at high frequency. With further optimization, this method could enable fine-scale characterization of formation properties, providing a basis for drilling parameter optimization, wellbore trajectory adjustment, and subsequent hydraulic fracturing design. Therefore, real-time lithology identification based on near-bit vibration signals is important for improving drilling efficiency, mitigating downhole risks, and enabling efficient reservoir development.

This study developed a lithology identification model based on laboratory experimental data. It preliminarily verified the feasibility of using near-bit vibration features combined with machine learning algorithms to identify changes in rock formation properties. However, external interfering factors have not yet been taken into account. For example, the effects of confining stress, pore pressure, and drilling fluid on vibration have not yet been taken into account. Moreover, the sample size used for model construction is currently limited and should be expanded. Future studies should include field validation and integrate multi-source information, such as WOB, torque, and rotational speed, to improve the accuracy and generalizability of real-time lithology identification.

## 4. Conclusions

In this paper, a test rig that replicates the field drill rig structure is built. Rock breaking experiments using a full-size bit are carried out, and vibration signals are collected. These data are subjected to analysis using both time domain and frequency domain approaches. The effects of lithology and drilling parameters on the vibration signals are investigated. Multiple artificial intelligence algorithms are used to establish a lithology discrimination model, enabling the use of vibration signals to discriminate lithology classes.

(1) Within the range of the experimental parameters used in this study, drilling parameters mainly affect the vibration amplitude, whereas the frequency composition is mainly affected by lithology. As rock mass strength increases, the drill bit vibration amplitude rises, and the high-frequency component of the signal also increases. With the increase in WOB and rotational speed, the vibration signal amplitude of the drill string increases, the frequency composition exhibits little change, and the frequency amplitude increases.
(2) When drilling different lithologies, the vibration signals of the drill bit exhibit quantifiably distinct characteristics, which can be characterized by extracting several key features. The t-SNE algorithm is used for cluster analysis to realize the effective discrimination of rock strata, and the validity of the selected features is verified.
(3) The vibration response at the drill bit achieves excellent performance in identifying lithology classes. The BP neural network, random forest, and GRNN models are used to establish the rock sample recognition model, respectively, and the results showed that the test accuracies are 97.4%, 96.4%, and 95.2%, respectively.

## Figures and Tables

**Figure 1:**
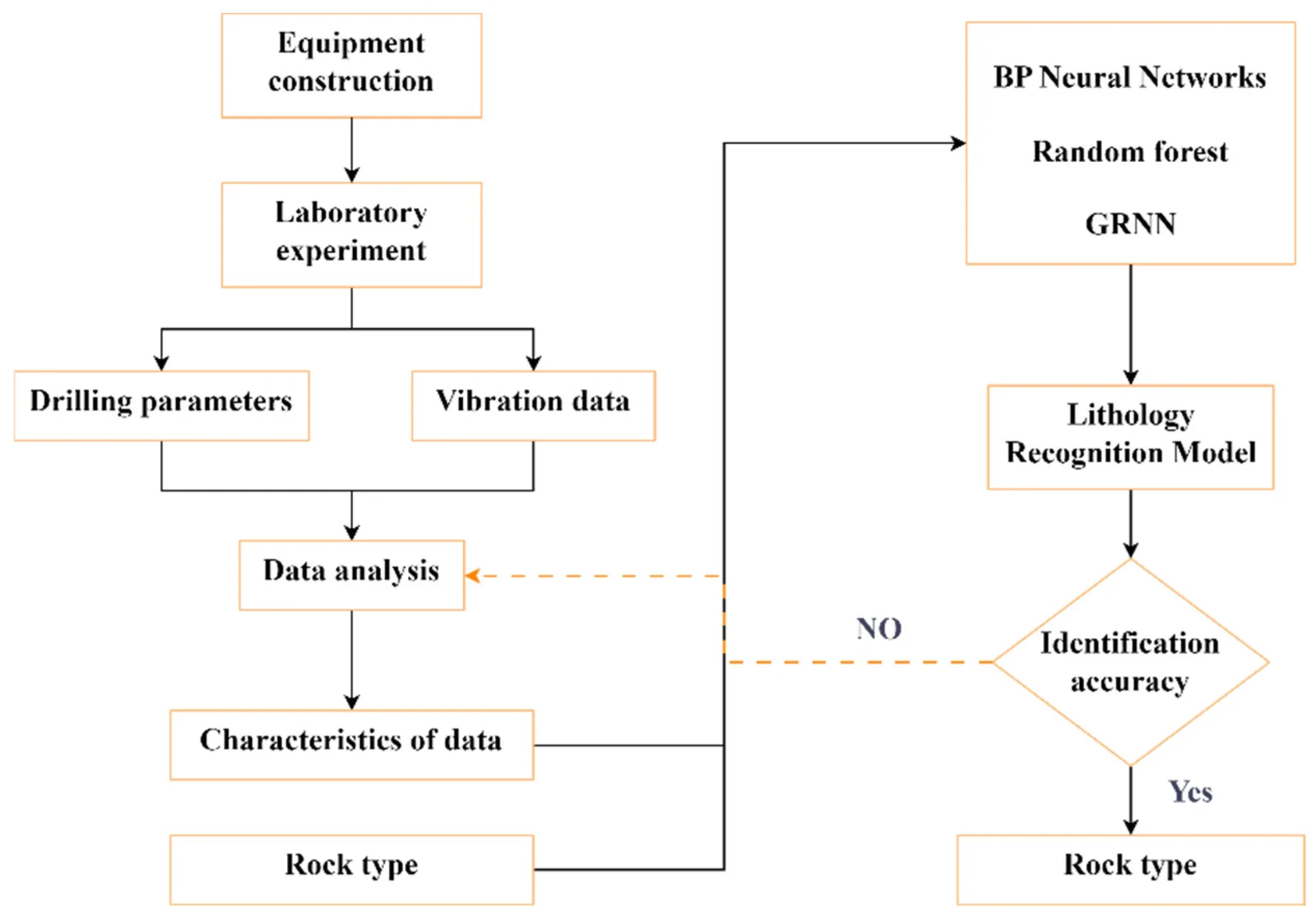
Technical flow chart for modeling.

**Figure 2:**
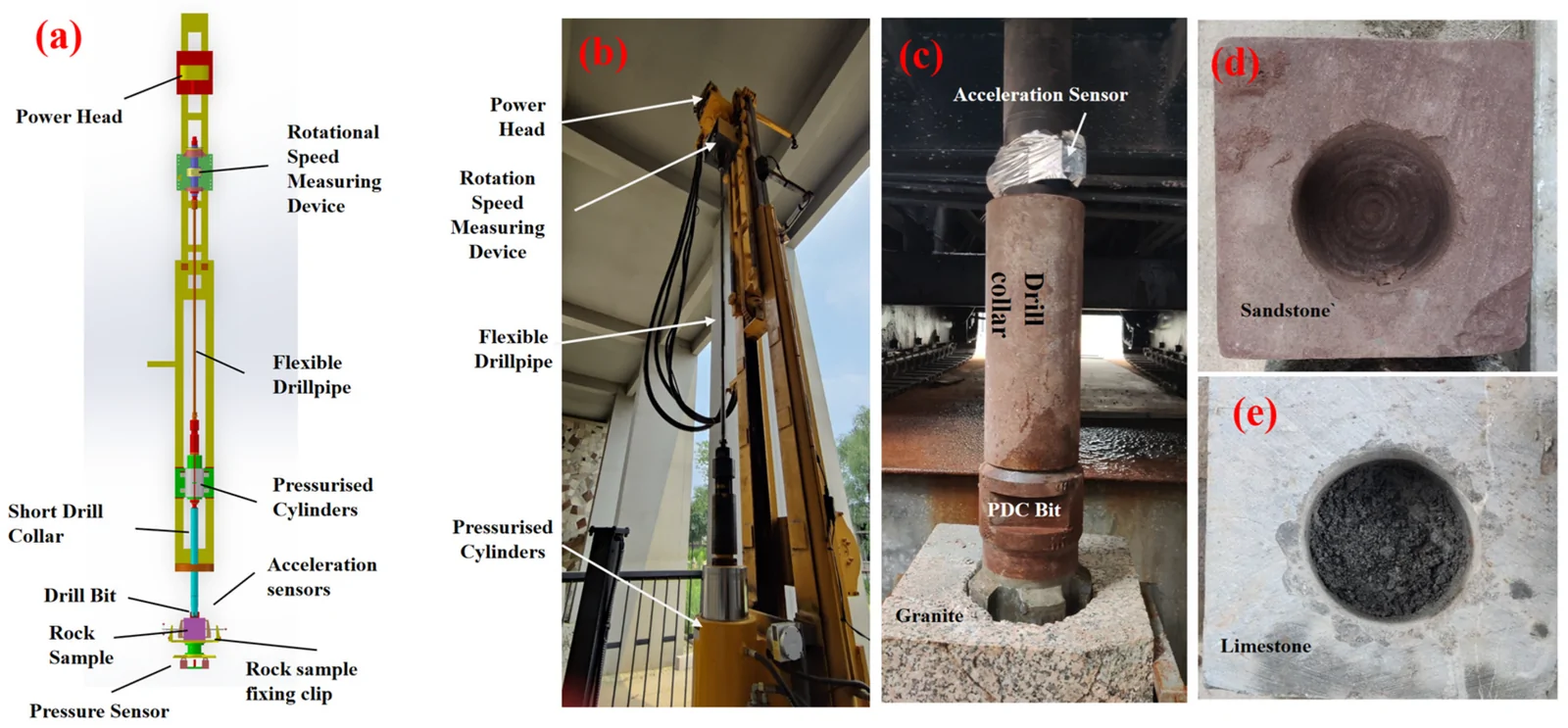
Laboratory experiments and rock samples. (**a**) Schematic diagram of the experimental setup. (**b**) Upper device of the drilling rig. (**c**) Bottom device of the drilling rig and granite sample. (**d**) Sandstone sample after drilling. (**e**) Limestone sample after drilling.

**Figure 3:**
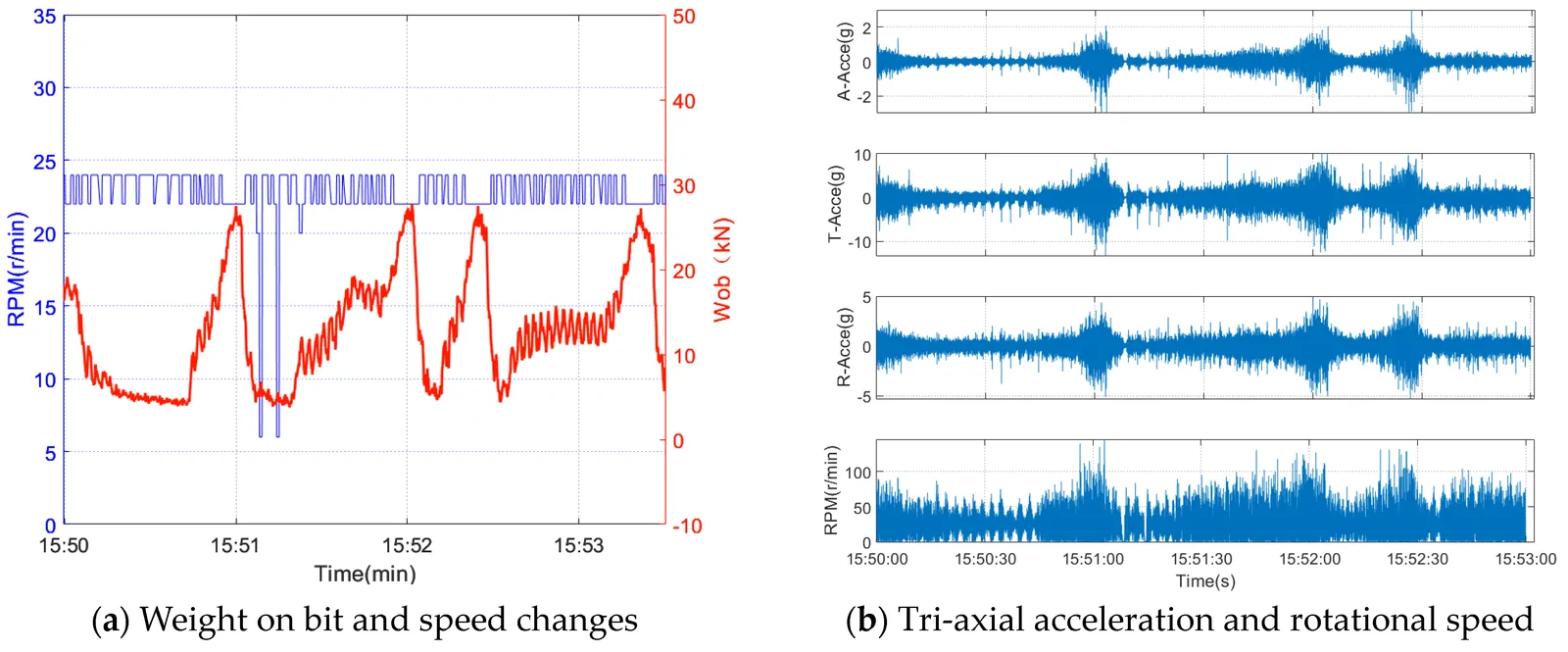
Drilling parameters and vibration data when drilling limestone.

**Figure 4:**
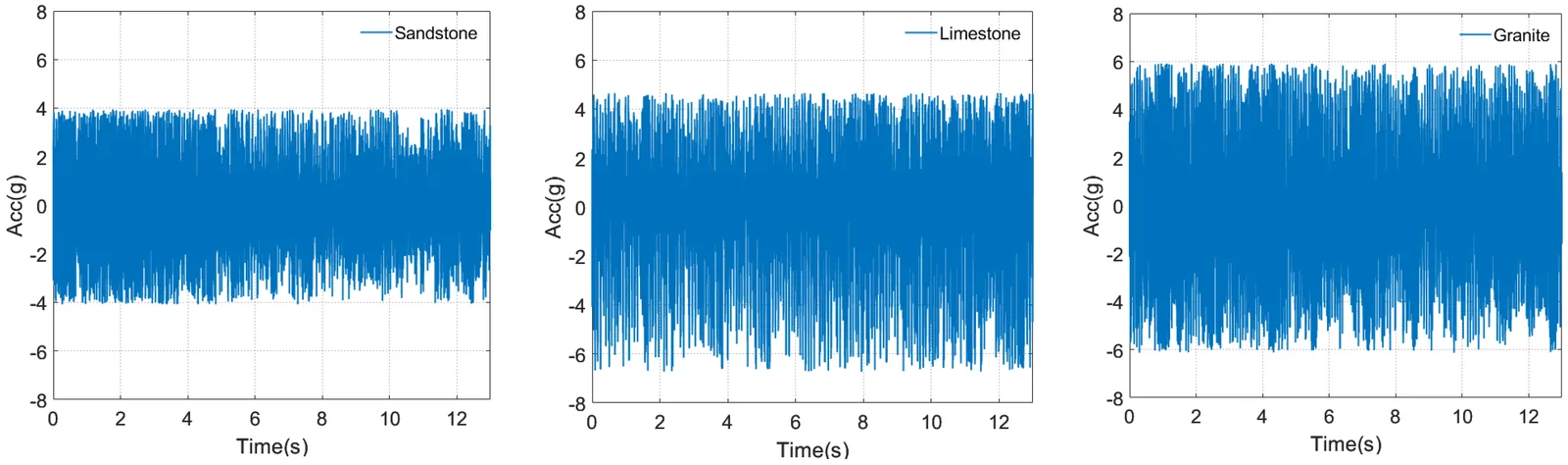
Three-rock-sample drilling acceleration signals.

**Figure 5:**
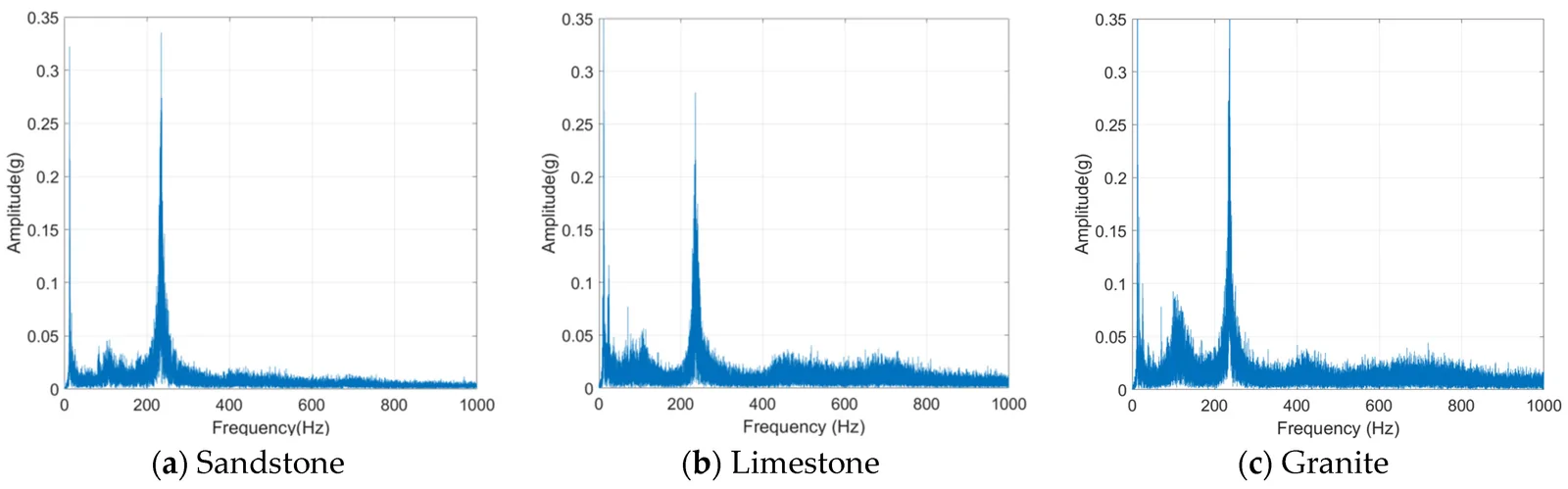
Spectrogram of three accelerations.

**Figure 6:**
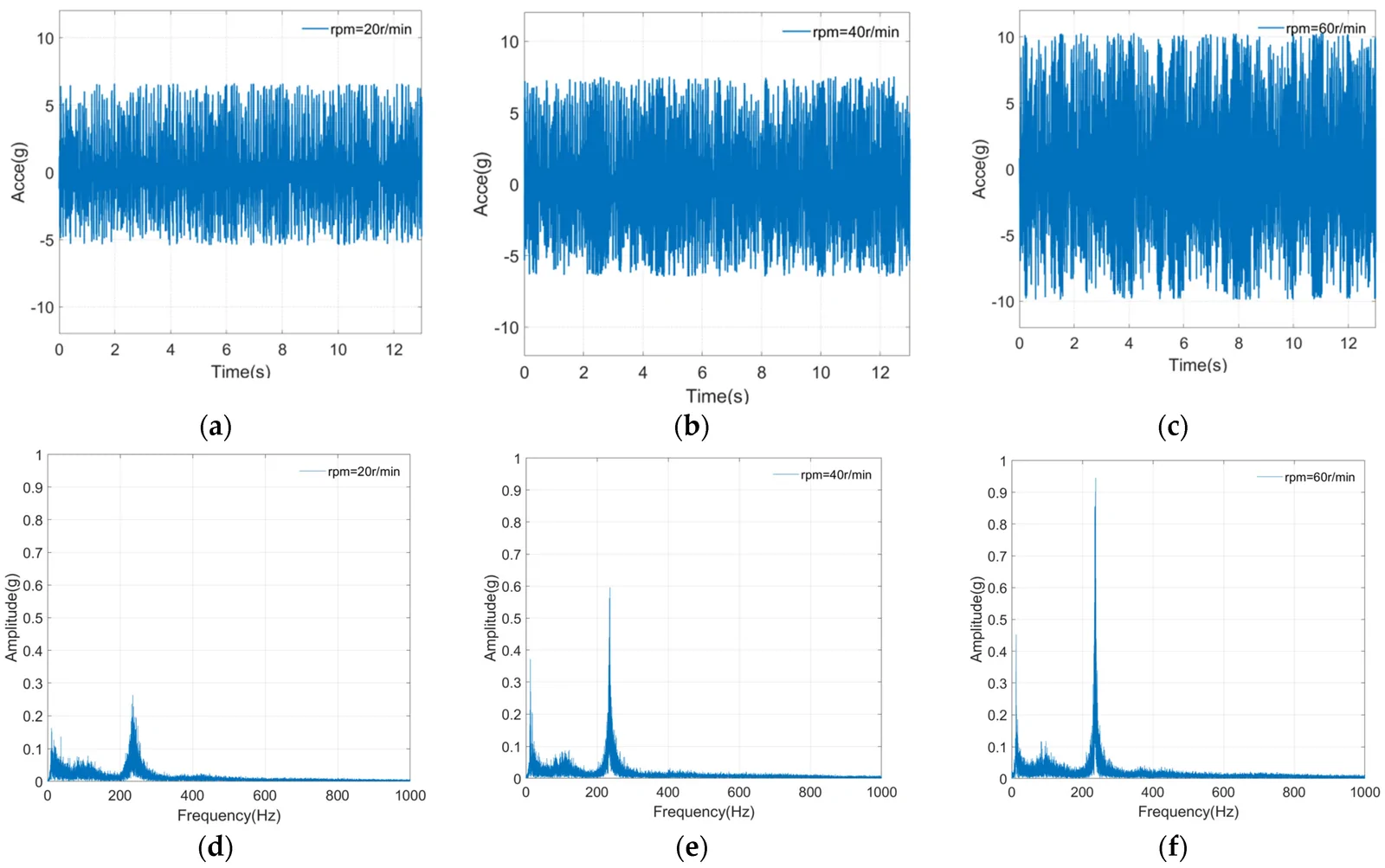
Comparison of tangential acceleration under three rotating speed conditions. (**a**) Tangential acceleration at 20 r/min. (**b**) Tangential acceleration at 40 r/min. (**c**) Tangential acceleration at 60 r/min. (**d**) Spectral diagram of tangential acceleration at 20 r/min. (**e**) Spectral diagram of tangential acceleration at 40 r/min. (**f**) Spectral diagram of tangential acceleration at 60 r/min.

**Figure 7:**
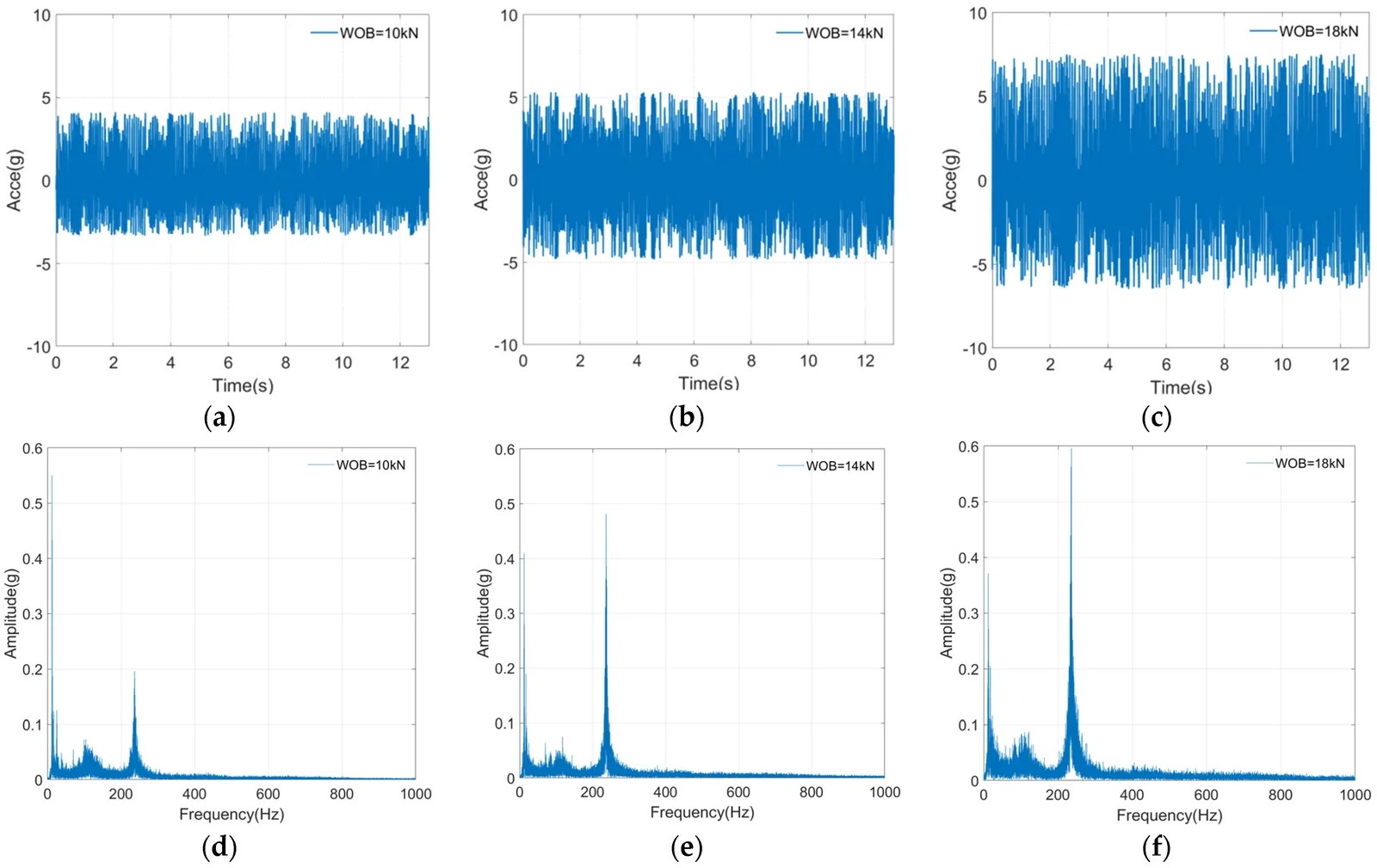
Comparison of tangential acceleration under three different bit weight conditions. (**a**) Tangential acceleration at 10 kN. (**b**) Tangential acceleration at 14 kN. (**c**) Tangential acceleration at 18 kN. (**d**) Spectral diagram of tangential acceleration at 10 kN. (**e**) Spectral diagram of tangential acceleration at 14 kN. (**f**) Spectral diagram of tangential acceleration at 18 kN.

**Figure 8:**
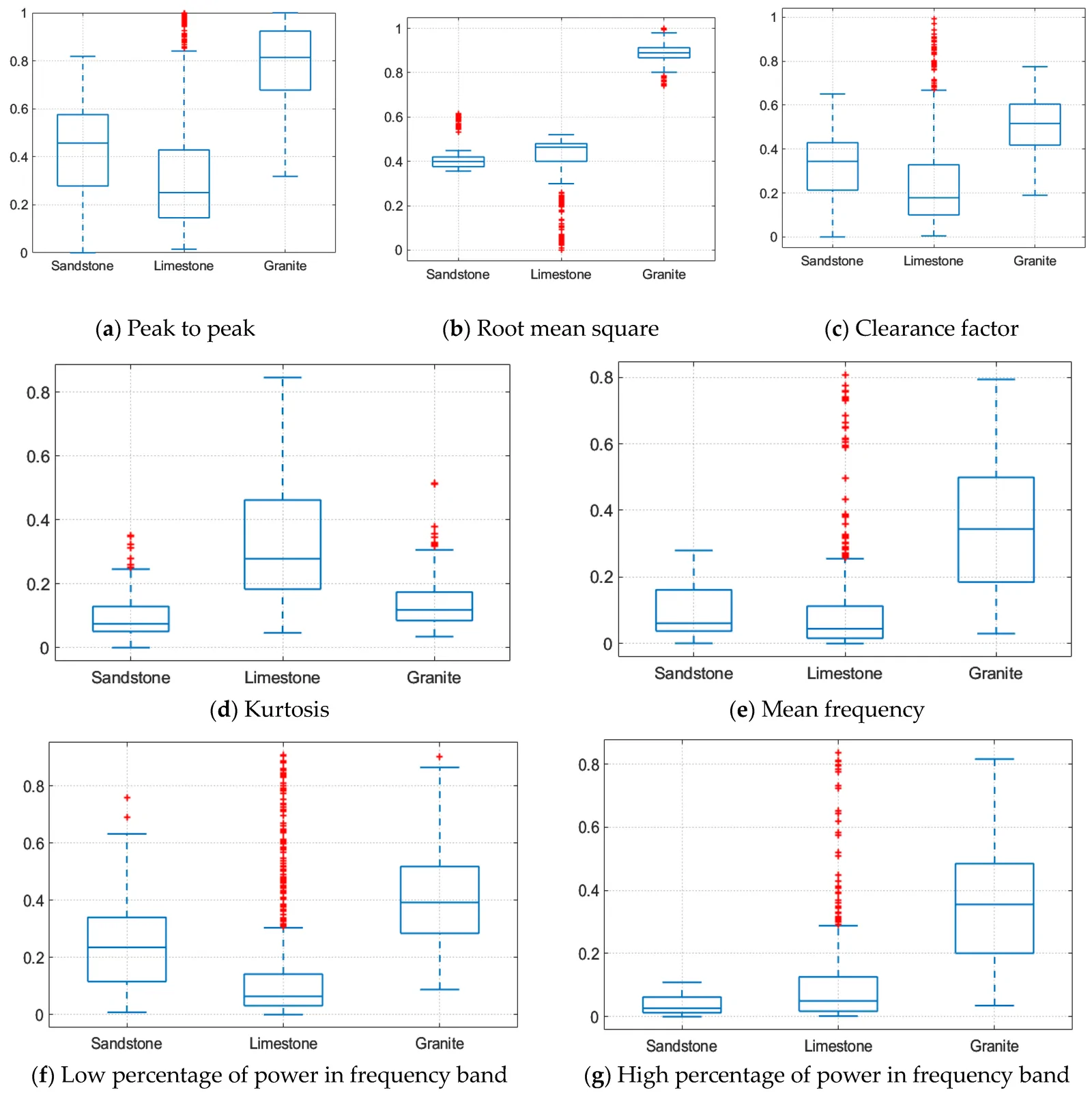
Box plots of normalized data features.

**Figure 9:**
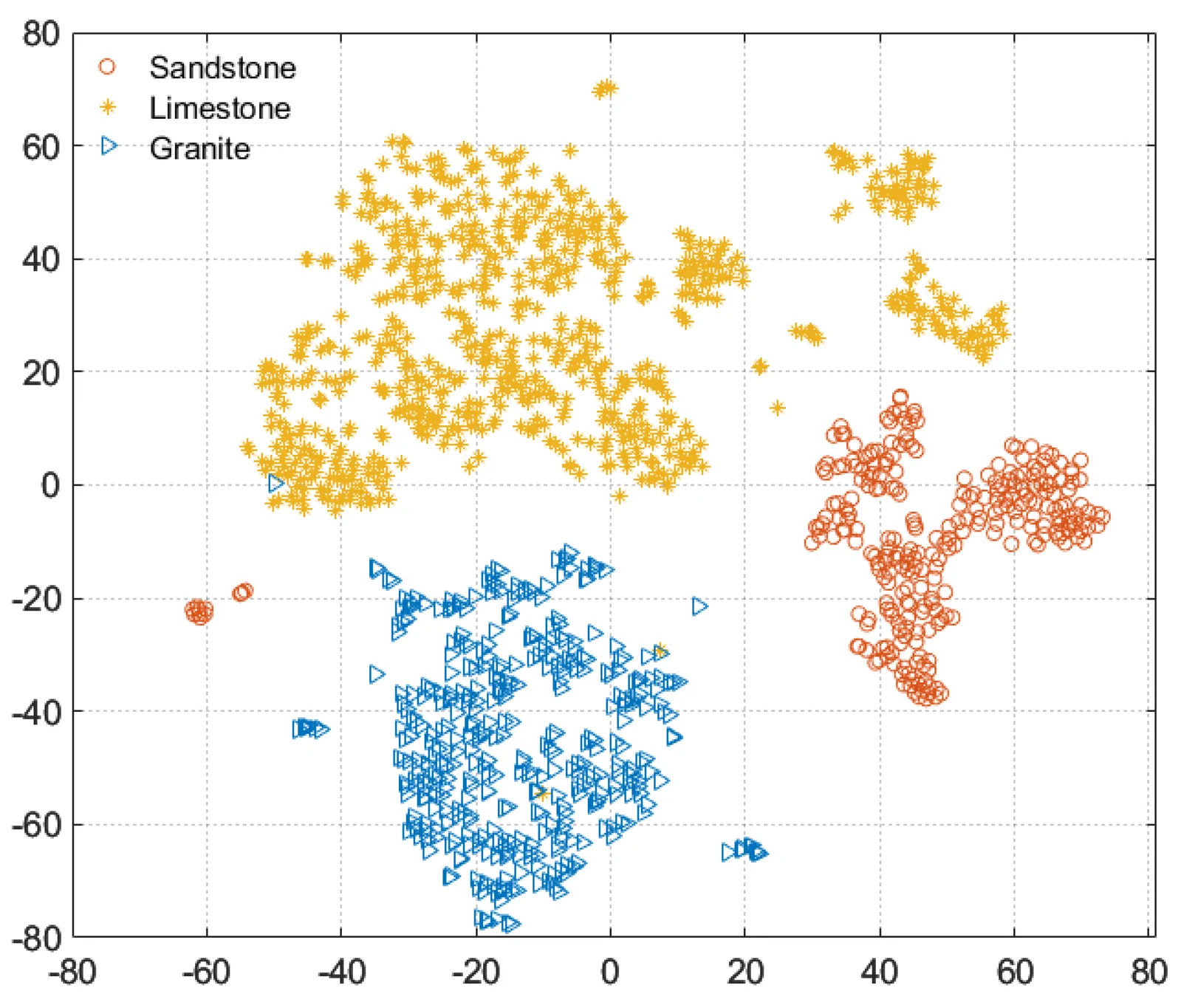
Data feature visualization based on t-SNE algorithm.

**Figure 10:**
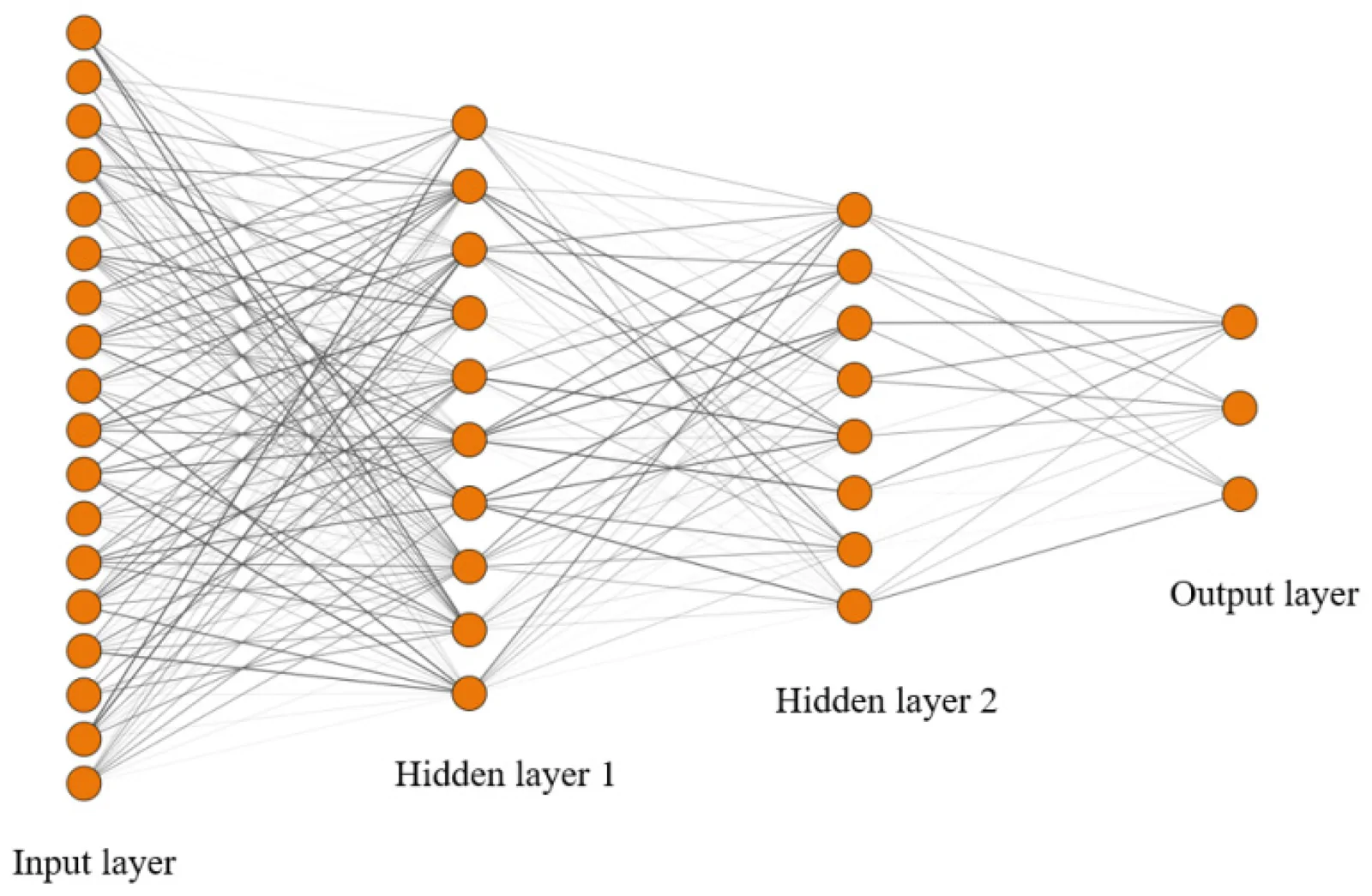
Schematic diagram of the back propagation (BP) neural network architecture.

**Figure 11:**
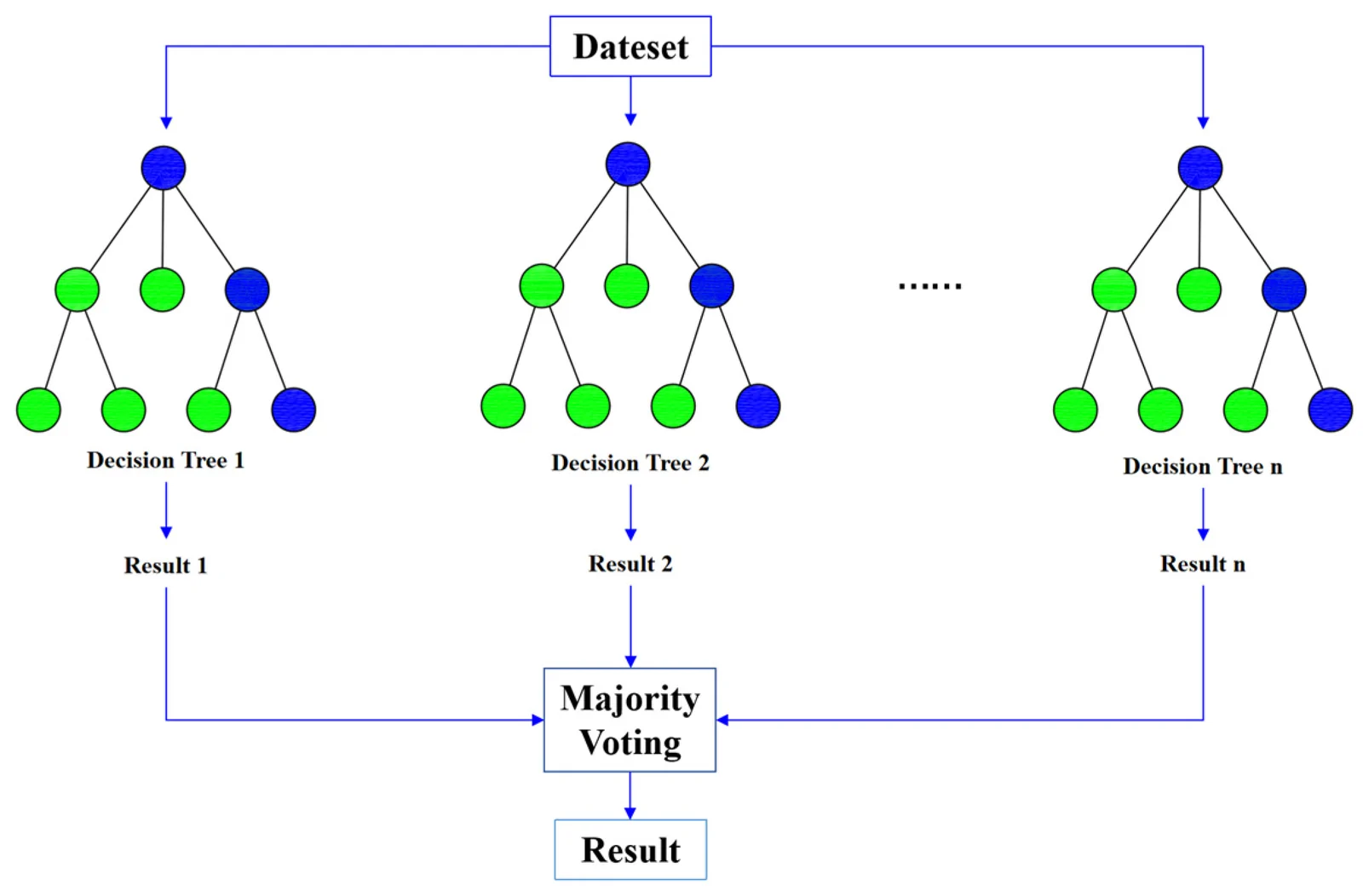
Schematic diagram of random forest.

**Figure 12:**
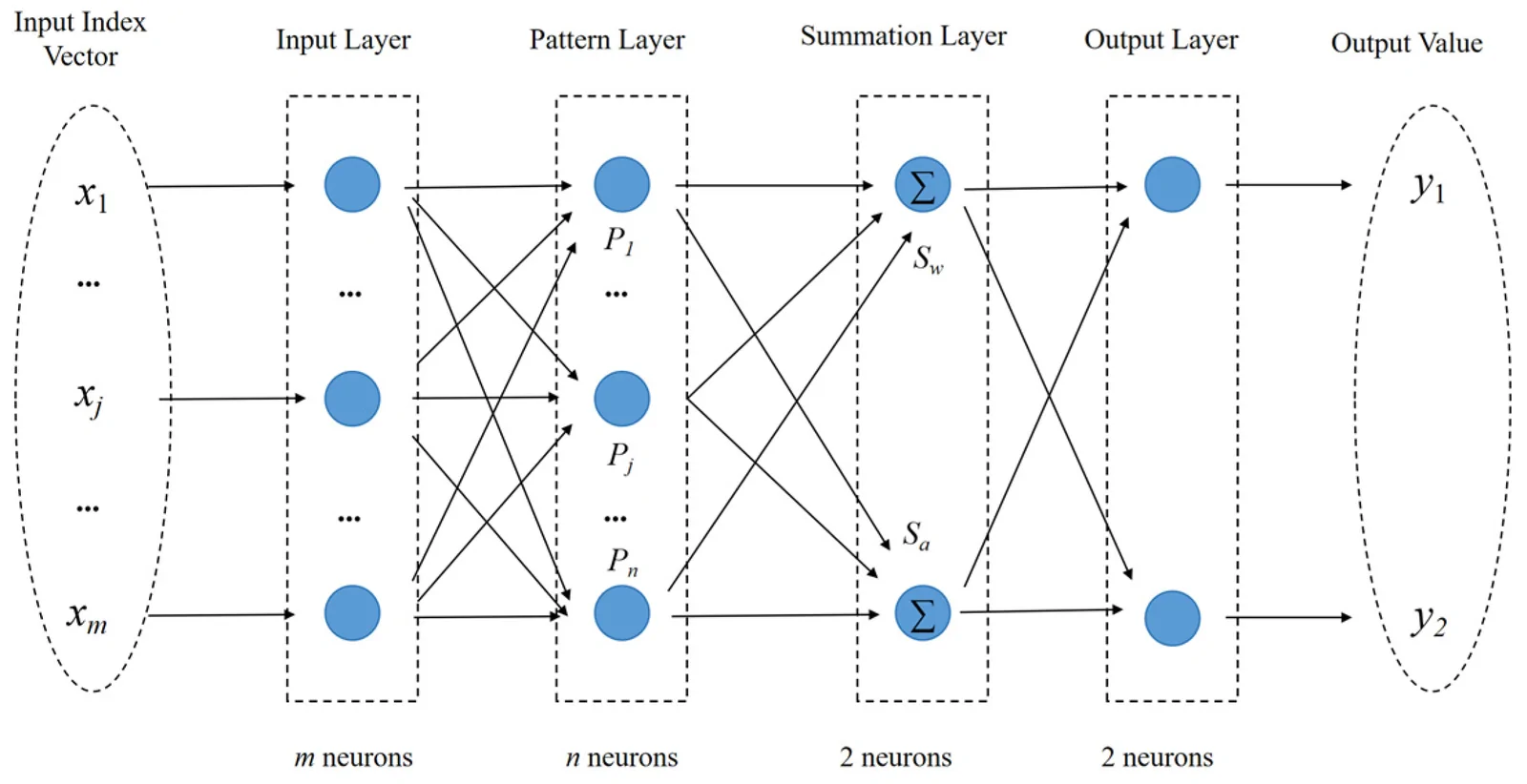
Topological structure of GRNN model.

**Figure 13:**
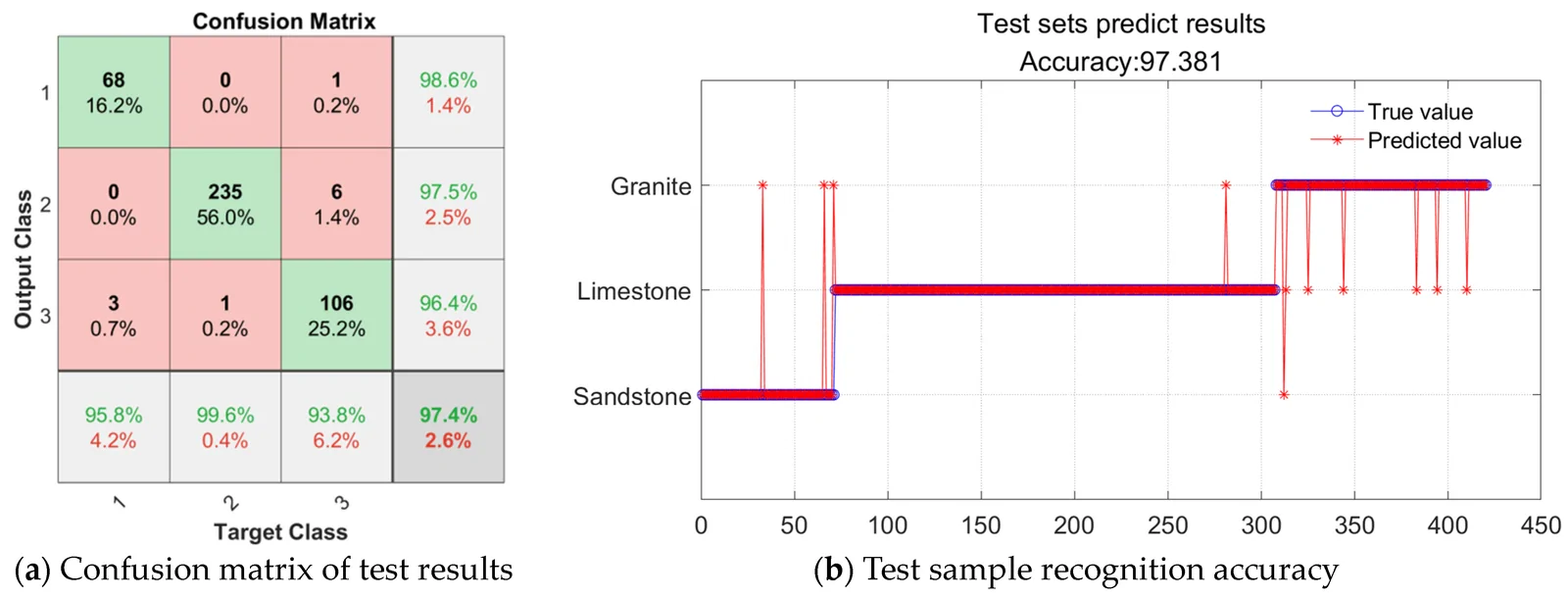
Identification accuracy and results of lithology identification model.

**Figure 14:**
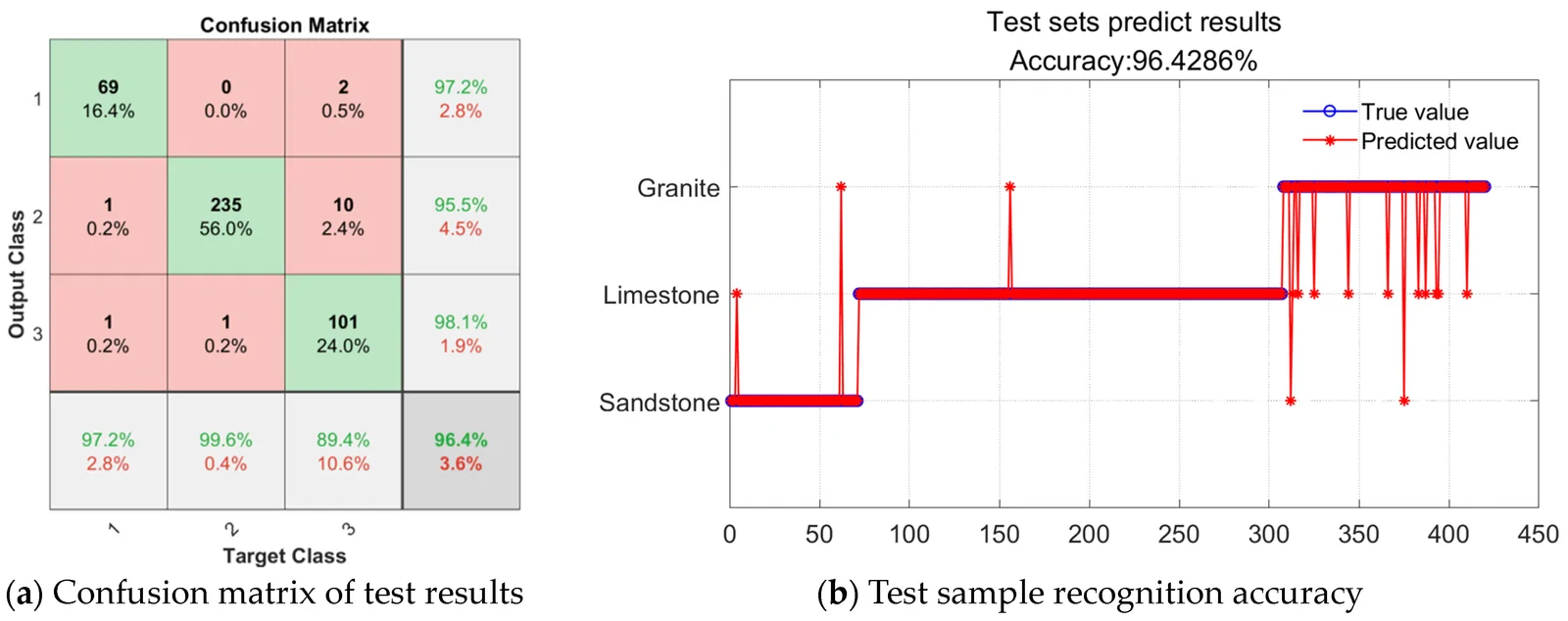
Identification accuracy of lithology identification model established by random forest.

**Figure 15:**
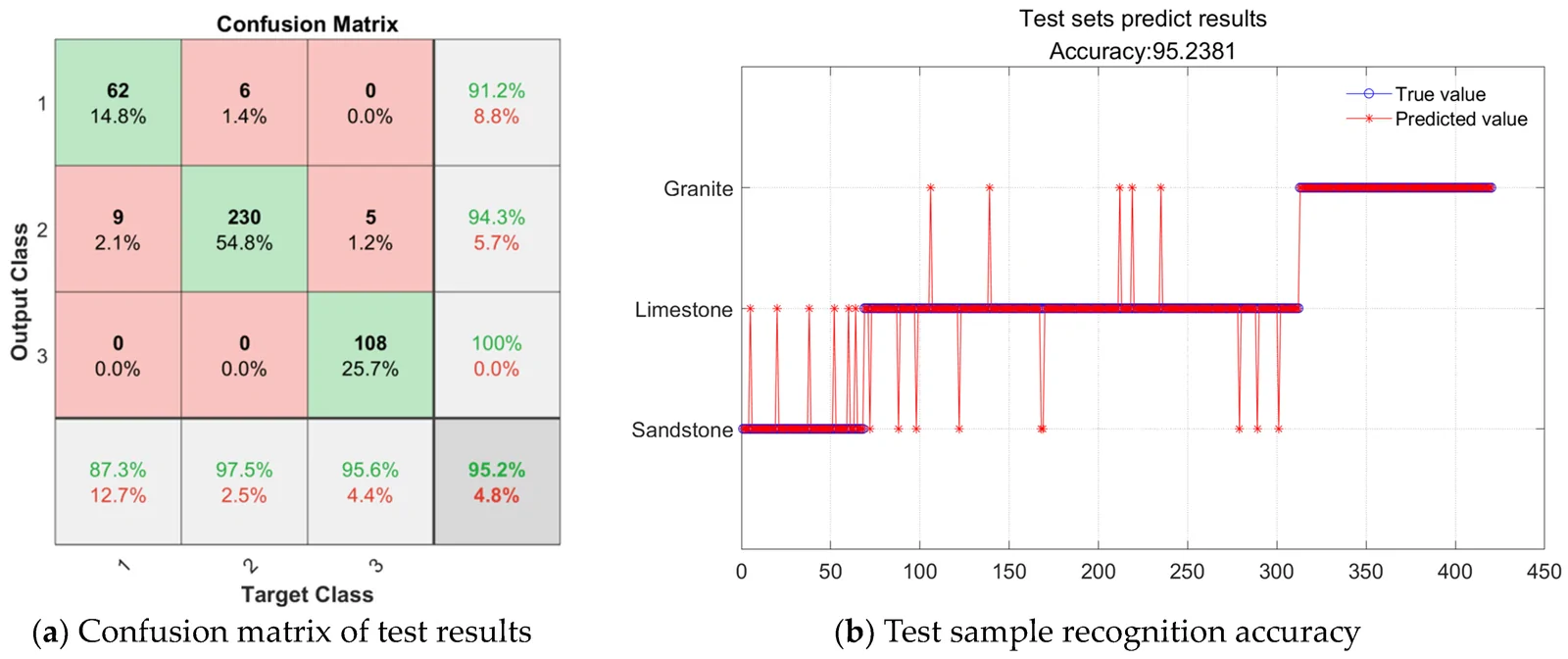
Identification accuracy of lithology recognition model based on GRNN.

**Table 1:** Summary of the experimental parameters.

No.	Rock Sample	WOB(kN)	Rotational Speed (r/min)
1	sandstone, limestone and granite	15	40
2	granite	20	20, 40, 60
3	granite	10, 14, 18	40

**Table 2:** The characteristic value of the signal.

Data Type	Analytical Method	Feature Type	Equation
Axial acceleration Tangential acceleration Rotational speed	Time domain	Root mean square	$RMS=\sqrt{\frac{1}{N}\sum_{i=1}^{N}x_{i}^{2}}$
		Clearance factor	$C_{e}=\frac{x_{peak}}{{\left(\frac{1}{n}\sum_{i=1}^{n}\sqrt{\left|x_{i}\right|}\right)}^{2}}$
		Kurtosis index	$k=\frac{E[{\left(x-u\right)}^{4}]}{\sigma^{4}}$
		Peak to peak	$x_{peak}=x_{max}-x_{min}$
Axial acceleration Tangential acceleration	Frequency domain	Mean frequency	$FC=\frac{\int_{0}^{+\infty }fP(f)df}{\int_{0}^{+\infty }P(f)df}$
		Percentage of power in frequency band	$ratio=\frac{\int_{a}^{b}P(f)df}{\int_{0}^{+\infty }P(f)df}$
Drilling Parameter	Weight on bit, Rotational speed		

**Table 3:** Algorithm types and model parameters.

Algorithm Type	Algorithms and Hyperparameter	Value
BP	Training	Scaled conjugate gradient
	Hidden layers	3
Random forest	Number of decision trees	50
	Minimum number of samples for leaf nodes	3
GRNN	Spread	0.3

## Data Availability

The data supporting the findings of this study are available from the corresponding author upon reasonable request.
